# An Open-Source Add-On EVOM^®^ Device for Real-Time Transepithelial/Endothelial Electrical Resistance Measurements in Multiple Transwell Samples

**DOI:** 10.3390/mi12030282

**Published:** 2021-03-08

**Authors:** Bibek Raut, Li-Jiun Chen, Takeshi Hori, Hirokazu Kaji

**Affiliations:** 1 Department of Finemechanics, Graduate School of Engineering, Tohoku University, 6-6-01 Aramaki, Aoba-ku, Sendai 980-8579, Japan; bibek.raut03@gmail.com (B.R.); lij.c29@gmail.com (L.-J.C.); takeshi.hori.b5@tohoku.ac.jp (T.H.); 2 Department of Biomedical Engineering, Graduate School of Biomedical Engineering, Tohoku University, 6-6-01 Aramaki, Aoba-ku, Sendai 980-8579, Japan

**Keywords:** real-time TEER measurement, multi-array switching, tight junction, EVOM^®^, chopstick electrode, 4-electrode resistance, ohmic resistance

## Abstract

This study provides design of a low-cost and open source add-on device that enhances the functionality of the popular EVOM^®^ instrument for transepithelial/endothelial electrical resistance (TEER) measurement. The original EVOM^®^ instrument is designed for measuring TEER in transwell samples manually using a pair of Ag/AgCl electrodes. The inconsistency in electrode placement, temperature variation, and a typically large (12–24 h) time interval between measurements result in large data variabilities. Thus, to solve the current limitation of the EVOM^®^ instrument, we built an add-on device using a custom designed electronic board and a 3D printed electrode holder that allowed automated TEER measurements in multiple transwell samples. To demonstrate the functionality of the device prototype, we monitored TEER in 4 transwell samples containing retinal cells (ARPE-19) for 67 h. Furthermore, by monitoring temperature of the cell culture medium, we were able to detect fluctuations in TEER due to temperature change after the medium change process, and were able to correct the data offset. Although we demonstrated the use of our add-on device on EVOM^®^ instrument only, the concept (multiplexing using digitally controlled relays) and hardware (custom data logger) presented here can be applied to more advanced TEER instruments to improve the performance of those devices.

## 1. Introduction

Epithelial and endothelial cells line the surface of the body. Adjacent cells of both epithelial and endothelial cells are connected to each other via intercellular junction called tight junction that regulates the diffusion of molecules and ions between the apical and basolateral side of the body [1]. Quantification of this barrier is important to evaluate the suitability of in vitro cellular barrier models for drug toxicity [2,3,4] or transport studies [5,6,7].

Transepithelial/endothelial electrical resistance (TEER), a quantitative technique to assess electrical resistance of the cellular monolayer during culture has become one of the most popular quantitative techniques to evaluate the integrity of tight junction [8]. The major advantage of the TEER technique over traditional permeability measurements is that it is quick, label-free, and non-invasive [8,9]. As such, the TEER technique has been widely used to evaluate in vitro models such as the blood-brain barrier [10,11,12], the intestine epithelial barrier [13,14], the alveolar epithelial barrier [15,16], and the blood-retina barrier [17,18,19].

TEER instruments can be categorized into mainly two types: volt-ohmmeter and impedance spectroscope [8]. Volt-ohmmeter is a basic type of instrument where a constant alternating current (AC) is supplied through the sample and the resulting voltage drop is measured. Impedance spectroscope on the other hand measures the resulting current drop at a range of AC frequency signals [9,20,21]. Although impedance spectroscopes give better results, these instruments are relatively expensive. Many research labs therefore still use the simple volt-ohmmeters to measure TEER.

**Figure 1:**
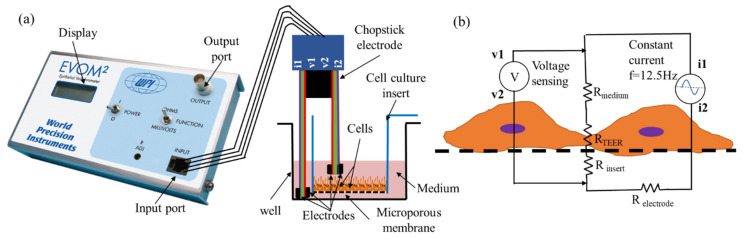
Transepithelial/endothelial electrical resistance (TEER) measurement using EVOM^®^ instrument. (**a**) A typical experiment setup for a four-electrode resistance measurement of a cellular monolayer in a transwell cell culture insert. (**b**) A simplified equivalent circuit diagram to model TEER measurement in a tissue culture transwell sample. EVOM^®^ supplies constant AC current through i1 & i2 electrodes, and measures resulting voltage drop through v1 & v2 electrodes to calculate total electrical resistance constituting of the cell layer R_TEER_, the cell culture medium R_medium_, the microporous membrane insert R_insert_, while eliminating resistance of the electrode medium interface R_electrode_.

One such popular volt-ohmmeter is the EVOM^®^ (World Precision Instruments (WPI), Sarasota, FL, USA)-. It is widely utilized for measuring TEER in transwell samples [8]. It comes with a chopstick type electrode with four electrode terminals that are inserted manually across a transwell insert (Figure 1a). By passing a constant AC current through the outer electrodes and measuring the voltage drop across the inner electrodes, the ohmic resistance of the tissue barrier sample is calculated (Figure 1b). The resistance value is recorded, usually at an interval of 24 h, and the TEER value is calculated by subtracting the resistance of the tissue barrier from the initial background value (freshly seeded insert samples). Hatherell et al. used EVOM^®^ to evaluate barrier integrity of blood brain barrier [22] and Chen et al. used it to investigate ARPE barrier integrity as part of the investigation on choroidal angiogenesis [18].

Although, widely utilized to quantify in-vitro barrier models, EVOM^®^ is limited to manual use only and the measurement can be influenced by many things. Two common factors include: incorrect alignment between electrodes and transwells, and temperature variation of the medium during measurement. Moreover, if the number of samples to be measured is large, it is hectic for the researcher to manually measure them. Also, the samples can only be taken out for TEER measurement few times in a day limiting the data record of number of observable changes in TEER. To solve these limitations of the EVOM^®^ instrument, an automated TEER instrument is desirable.

To measure TEER in real time, several commercial products such as cellZscope(Nano Analytics, Münster, Germany), TEER24 (Applied BioPhysics, Jordan Road Troy, NY, USA), and RMS AutoSampler (WPI) are available in the market. However, they cost thousands of dollars. As such, several research groups have built their own custom solutions to aid in TEER measurement. Theile et al. utilized a pair of multimeter to measure TEER in transwell samples [23]. Although this was a low-cost approach, a manual procedure was required to measure the TEER values. Further, Tu et al. used custom built gold plated electrodes [24] and Poenar et al. developed a low cost “sandwich ring” method [25] for real-time TEER measurements. However, time interval between measurement was at least 6 h and the measurement procedure was also manual which made it difficult to scale up.

Realizing these shortcomings, here we present an add-on device with two key features (1) automated real-time TEER measurement (15 min measurement interval) with in-situ temperature sensing and (2) TEER measurement in multiple sample with a single EVOM^®^ instrument. Our add-on device was able to (1) reduce the effect of environmental variables (2) increase number of observable TEER change data points (3) reduce burden on the researcher. The device prototype that we developed was built using open-source tools such as Arduino and off-the-shelf components that can be integrated with a widely used commercial EVOM^®^ volt-ohmmeter.

**Figure 2:**
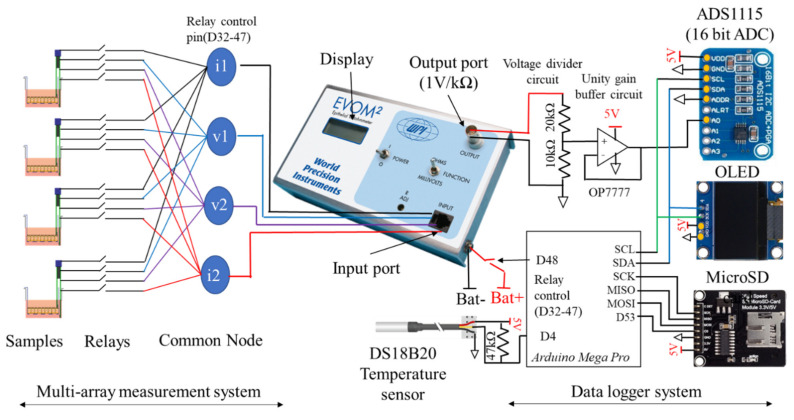
System schematic showing the multi-array measurement and custom data logger system of the proposed EVOM add-on device for real-time TEER measurement in multiple tissue culture transwell samples. Detail schematic is available in Supplementary File S1.

The add-on device consisted of two systems: the multi-array TEER measurement system and the custom designed data logger with a temperature sensor (Figure 2). Digitally controlled relays allowed inputs from a single chopstick electrode to be connected to the EVOM device sequentially one at a time. EVOM^®^ then measured the total resistance, feeding outputs to a custom-built data logger. The process is repeated until all samples were sequentially measured making it possible to automatically and sequentially measure resistance values from multiple samples using a single EVOM^®^ device. Finally, using the proposed add-on device, we demonstrated the TEER measurement in multiple samples of ARPE-19 cells in 12-well transwell inserts. In addition, to correct the effect of temperature variation, we monitored temperature of the cell culture medium which allowed further reduction in data variability.

## 2. Materials and Methods

### 2.1. Cell Culture and Staining

The human retinal pigment epithelial cell line (ARPE-19) was purchased from the American Type Culture Collection (ATCC, Manassas, VA, USA) and routinely sub-cultured in Dulbecco’s Modified Eagle Medium (DMEM) supplemented with 10% fetal bovine serum (FBS, S 1400–500, Biowest, Nuaillé, France) and 1% Antibiotic-Antimycotic (100×, 15240062, Gibco, Thermo Fisher Scientific, Waltham, MA, USA) at 37 °C in a humidified condition under 5% CO_2_. The passage number used for the experiments was 20–30. Prior to cell seeding on the 12-well cell culture insert (8 μm pore, #140656, Falcon, Tewksbury, MA, USA), cells cultured in T25 flasks (Corning #430639, Corning, NY, USA) were stained with green cell tracker (CellTrackerTM green CMFDA Dye, #C2925, ThermoFisher Scientific, Waltham, MA, USA) in serum free medium for 45 min following manufacturer’s protocol.

### 2.2. EVOM^®^ Add-On Device Design

#### 2.2.1. Custom Built Data Logger for EVOM^®^ System

Here, we utilized a popular TEER instrument EVOM^®^ (EVOM2, WPI) which is a 4-electrode type volt-ohmmeter with 1 Ω precision and 1–9999 Ω measurement range. It supplies a constant alternating current of 10 µA at 12.5 Hz through outer electrodes and measures the voltage drop across the inner electrodes as shown schematically in Figure 1b. Thus, EVOM measures the total ohmic resistance of the system utilizing 4 electrode resistance measurement technique. Four electrode technique that separates current-carrying and voltage measurement electrodes are essential as it nulls resistance of the electrode medium interface [19,23].

EVOM^®^ is designed for taking measurements from a single chopstick electrode at a time. The electrodes are dipped manually to measure the resistance of the tissue barrier in transwell samples. The resistance value is displayed on the LED screen. However, EVOM^®^ also contains a voltage output port that can be data-logged to record the data on a data logger. Here, we built one such data logger. EVOM^®^ outputs the measured resistance in the form of voltage (1 V per 1 kΩ) which were then fed to a voltage divider circuit. The maximum voltage output from the EVOM^®^ is 12 V however, the analog to digital converter (ADC) can only measure up to 5 V so a voltage divider circuit was used to increase the voltage measurement range. After that, the resulting voltage was fed to a unity buffer circuit and finally to the16-bit ADC (ADS1115) interfaced to an Arduino microcontroller (Arduino mega pro). An OLED display (0.96 inch OLED display) displayed the data while a microSD card module stored the data in a text file for future use.

#### 2.2.2. Multi-Array TEER Measurement System

Although EVOM^®^ allows real-time TEER measurement using external data logger, it can only be connected to a single electrode set at any time. This limited capability means we need same number of EVOM^®^ instruments for the number of samples we want to measure. Thus, to allow for multiple measurements using a single device, we used digitally controllable relays (switch). As shown in Figure 2, at one particular point in time, only one switch was turned on. After measurement (typically 15 s), the switch was turned to another sample and so forth until all samples were sequentially measured. This switching technique allowed multiple samples to be measured using single device. All wires from the electrodes were interfaced through a relay. It was then fed to a common node terminal (i1, i2, v1, v2). Then these common node terminals were connected to the input port of the EVOM^®^ instrument. Here, 4 samples have been used for proof-of-concept demonstration. First, all relays connecting to first sample was turned on. EVOM^®^ measured the resistance from this sample. After that, relays of the first sample were disconnected and relays of second samples were turned on and the resistance of that sample was measured. Likewise, relays were then switched to next samples until resistance of all samples were sequentially measured. Here, each sample was measured for 15 s and the resistance values measured during this time interval were averaged for each sample. Lastly, after measuring the last sample, EVOM^®^ was turned off for 14 min after which the measurement cycle was repeated.

#### 2.2.3. In-Situ Temperature Measurement

A waterproof temperature sensor (DS18B20, Amazon, Tokyo, Japan), was connected to digital pin 4 of the Arduino Mega microcontroller. The temperature sensor was dipped in the transwell containing equal volume of cell culture medium.

#### 2.2.4. Battery Power Management

The internal battery of EVOM^®^ has limited working range. It is originally powered by 2700 mAH, 6 V NiMH battery. However, to charge EVOM, 12 V DC power adapters are used. According to manufacturer’s datasheet, it is recommended to not use the device while it is charging, as it is sensitive to noise from the AC power outlets. Since 2700 mAH can power the device for only few hours, it was replaced with 7.2 V 4200 mAH LiPo battery (LiPo battery, Amazon). The connection of external battery to EVOM instrument is shown in Figure S1. Although not mentioned in the datasheet from WPI, EVOM could be powered by an external power source up to 12 V. Also, since the add-on device was programmed to take TEER measurement in a 15-min cycle (1 min total for 4 samples at 15 s interval + 14 min per measurement cycle), and the device was not used in between, the positive terminal was interfaced to a relay so that EVOM could be turned on only before taking the measurement. In addition, power to Arduino and relays were supplied separately via an external 5 V power adapter (5 V, 2 A DC Power Adapter, Amazon, Tokyo, Japan).

### 2.3. 3D Printed Holder Support for Electrode

**Figure 3:**
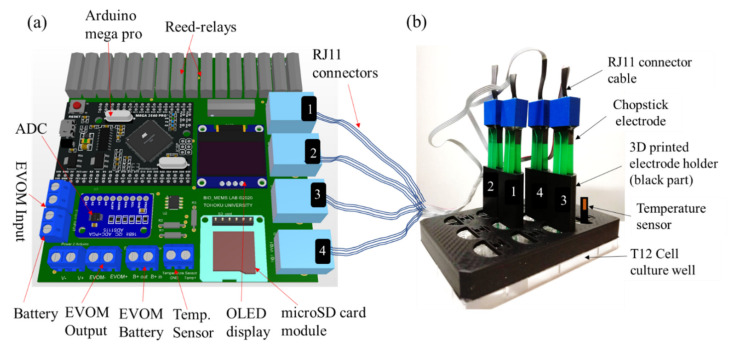
Add-on device with 3D printed electrode holder. (**a**) Printed circuit board (PCB) with electrical components of the add-on device. (**b**) Chopstick electrodes placed securely in the 3D printed electrode holder (showing 4 samples) and temperature sensor (wiring not shown). The add-on device was kept outside the cell culture incubator whereas the samples and the device were placed inside and connected through a flexible cable.

Since the distance between electrodes and its proper alignment is necessary to properly measure the TEER, a 3D printed case was used to securely place the chopstick electrodes. 3D CAD file for a chopstick holder was designed in SolidWorks2018 (Dassault Systèmes, Waltham, MA, USA) and 3D printed using Polylactic acid (PLA) material in a 3D printer (Qidi X-on2, Qidi Technology, Ruian, China) with a 0.4 mm diameter extrusion nozzle to align and keep the distance between chopstick electrodes the same. Figure 3a shows the printed circuit board (PCB) and Figure 3b shows an illustration of the commercial STX2/“chopstick” electrodes securely fitted in the 3D printed holder and put atop a 12-well plate (Falcon #353043). The holder was printed with a rim such that lateral movement of the 3D printed holder is prevented when put atop the 12 well plate. Figure S2 shows the detailed dimension of the 3D printed holder and Supplementary File S2 contains the CAD file for 3D printing. As a proof-of concept, only 4 electrodes were used, however for larger measurement samples, number of holders can be increased.

### 2.4. TEER Measurement Protocol Using EVOM^®^ Add-On Device

Following protocol was followed for TEER measurements:

(1) Seed each 12-well cell culture insert with 40,000 cell/cm^2^ mixed in 300 μL medium and incubate for 1 h to allow cells to attach.
(2) Replace the medium with 750 μL (insert), and 1.5 mL (well) fresh medium.
(3) Carefully insert the electrodes (housed in 3D printed holder) making sure the contact pads are fully immersed in the medium. Avoid electrode contact with insert’s membrane to avoid scratching attached cells. (longer electrode touches the bottom well ensuring consistency between samples)
(4) Put the 12-well plate and the electrodes carefully inside the incubator.
(5) Plug the RJ11 connectors from each electrode to the respective ports on the custom designed PCB board (Figure 3a).
(6) Insert microSD card in the SD-card slot, and press reset pin on Arduino to start taking data.
(7) To change medium, disconnect RJ11 connectors from the PCB and carefully take out the electrode set from 12-well plate.
(8) Replace medium with same volume as in step 2.
(9) Repeat step 3–8 (skip step 6).

### 2.5. TEER Data Collection

Data collected from three samples (inserts 1–3) containing cells and one sample without cells (blank) recorded on the microSD card were saved in a text format with nine sets of data (four sets of resistance data from each electrode, four sets of temperature data from a separate well containing medium exclusively for taking temperature data taken immediately after TEER measurement of each electrode, and one set of time data). Supplementary File S3 contains the Arduino program file. The text data were imported to Microsoft Excel 2013 (Microsoft corporation, Redmond, WA, USA) for further processing.

## 3. Results

### 3.1. Real Time TEER Measurement in Multiple Transwell Samples

**Figure 4:**
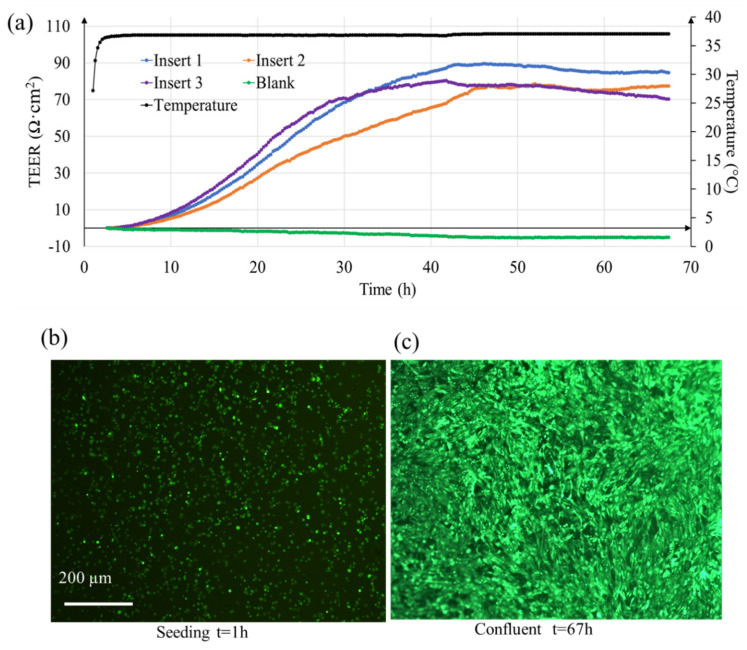
(**a**) Real-time TEER measurement of ARPE-19 cells (processed data) in transwell with in-situ temperature sensing. Inserts 1–3 contained cells and blank contained only cell culture medium. Fluorescence image of ARPE-19 cells (**b**) 1 h after seeding (**c**) 67 h when fully confluent.

TEER measured using EVOM^®^ systems is typically reported at an interval of 12–24 h. Since TEER values change continuously as the cells proliferate, much information is lost when the observation time interval is long. Here, as shown in Figure 4a, our system can report the TEER change at a much shorter interval of 15 min. In this proof-of-concept experiment with three cell containing samples (labeled as insert 1–3), it can be seen that as the cells proliferate, a gradual change in TEER occurs. However, this change is not same for all samples because the cell proliferation can differ in the transwell inserts. Nonetheless, as the cells become confluent, the TEER tend to plateau. The maximum average value here was (81 ± 7) ohms·cm^2^ (at t = 50 h, n = 3 samples) which is close to the previously reported TEER value of 90 ± 15 ohms·cm^2^ [18]. Figure 4b shows the fluorescence image of the ARPE-19 cells after 1 h of seeding, and Figure 4c shows cells when they were confluent (t = 67 h). For the non-cellular sample (labeled as blank), the resistance value remained constant with slight deviation (5 Ohms·cm^2^). This deviation is because we used a low-cost pseudo-Ag/AgCl electrode that comes with the EVOM^®^ system. The stability of the electrode can be improved further by using better quality reference electrodes.

### 3.2. Influence of Temperature and Other Variables in TEER Measurement

**Figure 5:**
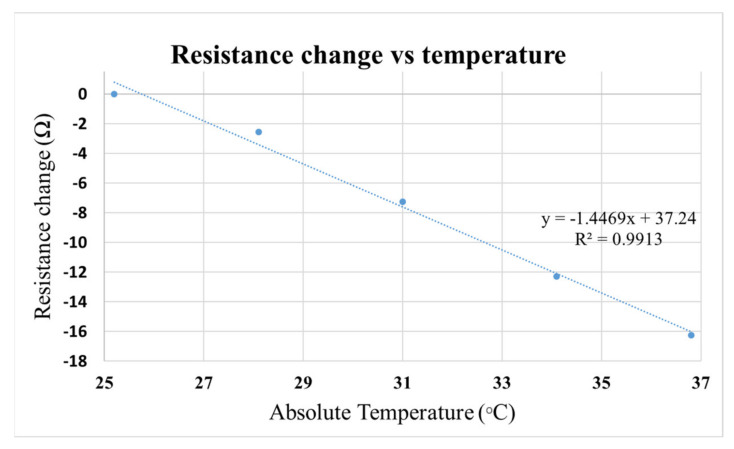
Graph showing the influence of temperature on resistance change of the cell culture medium.

Temperature can influence resistance reading. As shown in Figure 5, for every 1 °C rise in temperature, the resistance of the ARPE-19 cell culture medium decreased by 1.5 Ohms. TEER measured using EVOM^®^ system suffers from temperature deviation since the sample had to be brought outside the cell culture incubator for TEER measurement. However, with our add-on system, it was possible to measure TEER continuously inside an incubator, reducing data variation due to temperature change.

**Figure 6:**
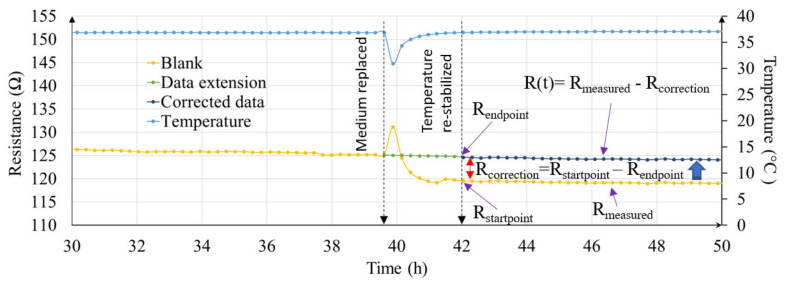
Graphical illustration showing influence in resistance after medium change process and procedure used to correct the data offset. Only “blank” data (t = 30 h to t = 50 h) is plotted for clarity.

Besides changes in temperature, TEER measurement can be influenced by other variables such as inconsistency in alignment of cell culture inserts and electrodes, and volume of cell culture medium. These variables are hard to predict and can play a big role in data inconsistency between different manual measurements. Figure 6, illustrates the real-time resistance values (yellow dotted line graph) in non-cellular medium only sample. At t = 39.5 h, medium was replaced. During this process, sample was taken outside the cell culture incubator and the medium was replaced with pre-warmed medium. The sample was then returned to the incubator. A sharp decrease in temperature followed by gradual rise after being placed in the incubator can be seen in Figure 6. However, at t = 42 h, even after temperature stabilizes to initial state, the resistance of the sample does not return to same value as would be expected. This is because during medium change process, the alignment between insert and electrode change, which results in a resistance offset. With a real-time resistance data coupled with temperature data, it was possible to then correct this data offset (green and dark blue dotted line graph in Figure 6). A detailed procedure of the data offset calculation is illustrated in Figures S4 and S5.

## 4. Discussion

The EVOM^®^ system lacks many functionalities compared to the more advanced TEER measurement instruments currently available in the market, yet it is popular and widely used for evaluating cellular barrier integrity in transwell cultured tissue samples [26,27]. However, EVOM^®^ instrument has many shortcomings. It is a basic type of volt-ohmmeter that needs to be used manually so the reliability of the data depends highly on the experience of the researchers conducting the experiment. This limitation on human caused errors on TEER measurement can be reduced if the device is automated. Thus, we developed a prototype add-on device to an EVOM instrument to enhance its functionality. Two key features of the add-on device included: (1) real-time data logging capability, and (2) multiple sampling with a single EVOM^®^ instrument. By utilizing custom built data logger and concept of multiplexing with digitally controllable relays, it was possible to have a smart and automated EVOM^®^ device, that as shown in Figure 6 and Figure S4 helped detect data offset caused by influence in temperature, electrode and insert positions.

Medium change process is unavoidable during cell culture. During medium replacement, the transwells have to be taken outside the cell culture incubator. This disturbs the continuous TEER measurement process. Also, (i) temperature change and (ii) movement of electrode during medium replacement process affects the TEER reading afterwards which adds a big challenge to the TEER measurement process [28,29]. The temperature-dependent changes of TEER values have been reported in some studies [30,31], and importantly, results with our system showed them clearly and chronologically (Figure 4 and Figure S4). The temperature dependence of TEER values should result from multiple mechanisms since the temperature changing may affect cells structurally and functionally as well as solution conductivity. It has been thought that TEER measurements should be conducted in an incubator at 37 °C because equilibration from 37 °C to room temperatures requires more than 20 min and it could be detrimental to cell functions [8]. Thus, measuring TEER while monitoring temperature of culture media should be a useful system in for example drug development with cell-based assays.

Here, we first quantified the effect of temperature on resistance change. Also, we used a 3D printed holder to secure the electrodes in place so that the distance between electrodes remained the same throughout the experiment. This is because the resistance measurement can change if the cell culture inserts are moved. During medium change process, the 3D printed holder with electrodes has to be removed, and the medium replaced. During that process, the cell culture inserts’ position is changed. This is one of the shortcomings in many manual measurements. By taking real-time measurement as presented in this paper, this data offset can be corrected. The data correction is further illustrate in Figures S4 and S5.

Although, there are many advanced TEER measurement systems with real-time data acquisition capabilities, they are costly [32,33]. As a result, EVOM^®^ is still widely used in many bioengineering labs. Here we have demonstrated that by integrating a data logger, multi-array relay module, and a temperature sensor, it is possible to enhance the capability of EVOM^®^ system and similar technique can be extended to further improve more advanced instruments in the market.

## 5. Conclusions

A prototype of an add-on device to the commercial EVOM^®^ instrument was demonstrated. The add-on device allowed real-time TEER measurement of multiple transwell samples using a single EVOM^®^ instrument. In this report, we (1) quantified the effect of temperature (25–37 °C) on TEER measurement, and corrected the resulting data offset, and (2) demonstrated real-time TEER measurement in multiple transwell samples. Thus, we demonstrated how TEER data fluctuation after regular medium change process could be detected and corrected. Further, we believe that the multiplexing concept using digitally controlled relays and electronic hardware (data logger) demonstrated here can be utilized in other more advanced TEER instruments as well.

## Supplementary Materials

The following are available online at https://www.mdpi.com/2072-666X/12/3/282/s1, please refer to the supplementary documents. Figure S1. External battery connection to EVOM S2. Drawing of 3D printed electrode holder, Figure S3. Schematic drawing of the electrical connections of the add-on-device, Figure S4. Graphical illustration of raw and processed TEER data, Figure S5. A copy of Figure 6 for further illustration of data correction after medium was replaced. Supplementary File S1: Schematic of the add-on device electronics, Supplementary File S2: CAD file for 3D printing chopstick holders, Supplementary File S3: Program code for the add-on device
