# Supplementary material for: An Open-Source Add-On EVOM® Device for Real-Time Transepithelial/Endothelial Electrical Resistance Measurements in Multiple Transwell Samples
Source: Micromachines (Basel). 2021 Mar 8;12(3):282. doi: 10.3390/mi12030282 (PMC8000980; doi:10.3390/mi12030282)
Supplement: Supplementary file 1 [file micromachines-12-00282-s001.zip › Supplementary_TEER_Paper.docx]

**Supplementary Document**

**An open-source add-on EVOM® device for real-time transepithelial/endothelial electrical resistance measurements in multiple transwell samples**

Bibek Raut ^1^, Li-Jiun Chen ^1^, Takeshi Hori ^1^, and Hirokazu Kaji ^1,2,^*

^1^Department of Finemechanics, Graduate School of Engineering, Tohoku University, 6-6-01 Aramaki, Aoba-ku, Sendai 980-8579 Japan;

^2^Department of Biomedical Engineering, Graduate School of Biomedical Engineering, Tohoku University, 6-6-01 Aramaki, Aoba-ku, Sendai 980-8579 Japan

*Correspondence kaji@tohoku.ac.jp;

Tel/Fax: +81-22-795-4249

**Contents:**

**Supplementary File S1:** CAD file for 3D printing chopstick holders. Only 4 chopstick electrodes were used as demonstration.

**Supplementary File S2:** Schematic of the add-on device electronics

**Supplementary File S3:** Program code for the add-on device. The code needs to be uploaded via Arduino software (<https://www.arduino.cc/>).

**Supplementary Figure S1:** External battery connection to EVOM

**Supplementary Figure S2:** Drawing of 3D printed electrode holder

**Supplementary Figure S3:** Schematic drawing of the electrical connections of the add-on-device

**Supplementary Figure S4:** Graphical illustration of raw and processed TEER data

**Supplementary Figure S5:** A copy of Figure 6 for further illustration of data correction after medium was replaced


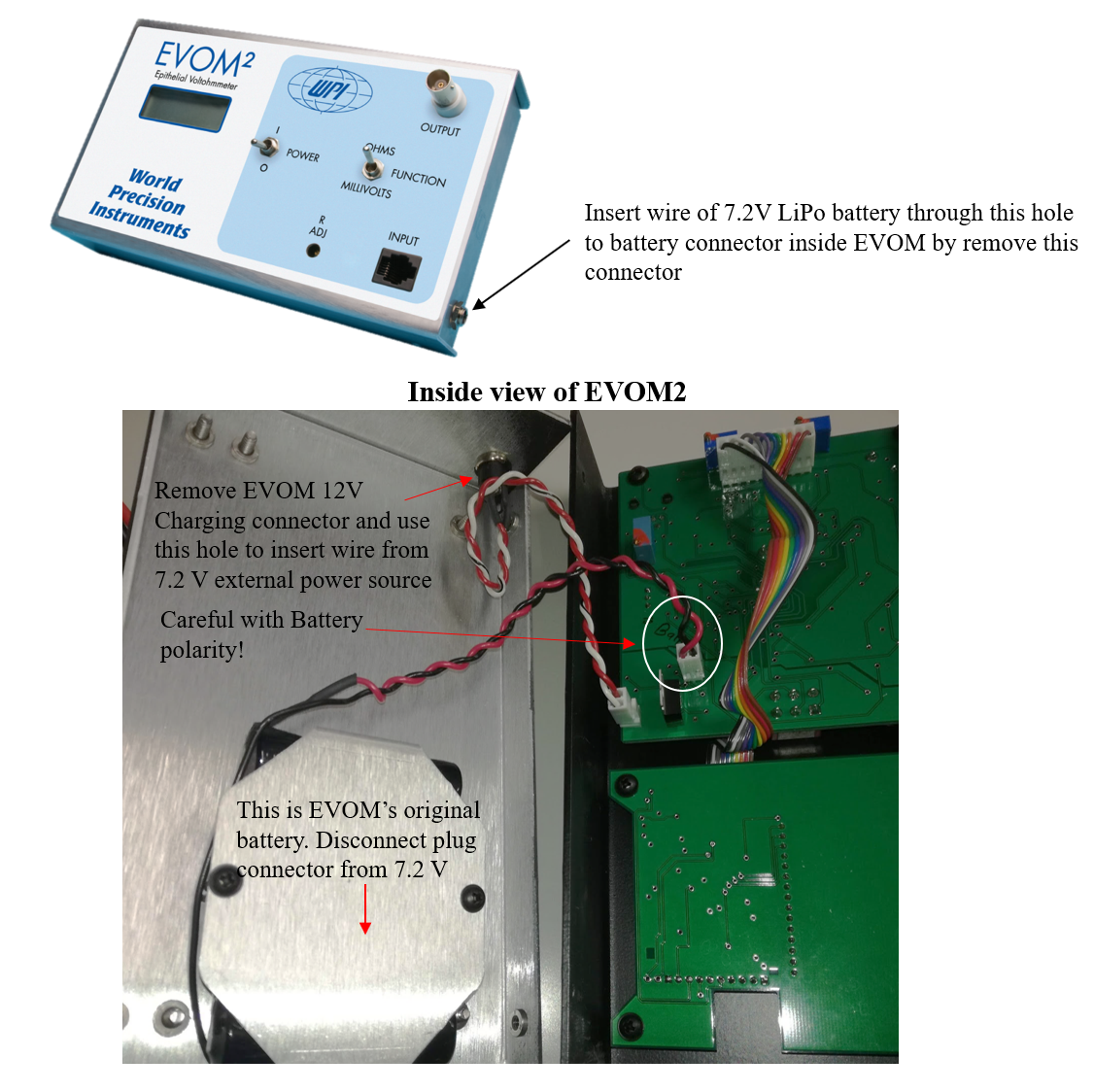


Figure S1. External battery connection to EVOM. Caution must be taken not to swap battery polarity otherwise, EVOM instrument could be damaged.


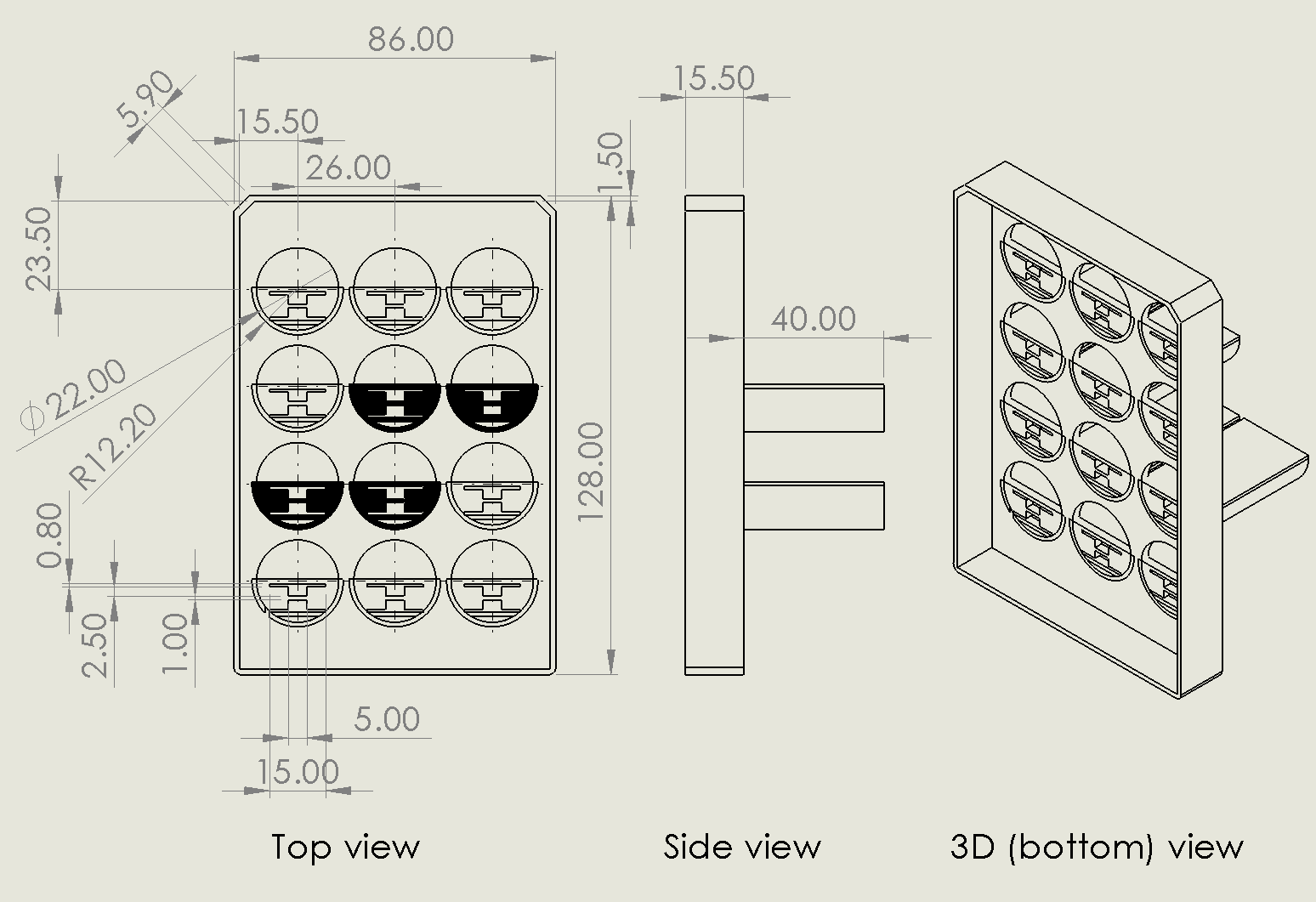


Figure S2. Drawing of 3D printed electrode holder. Only 4 holders were extruded and 3D printed as shown by dark area in the drawing. Dimensions are in mm.


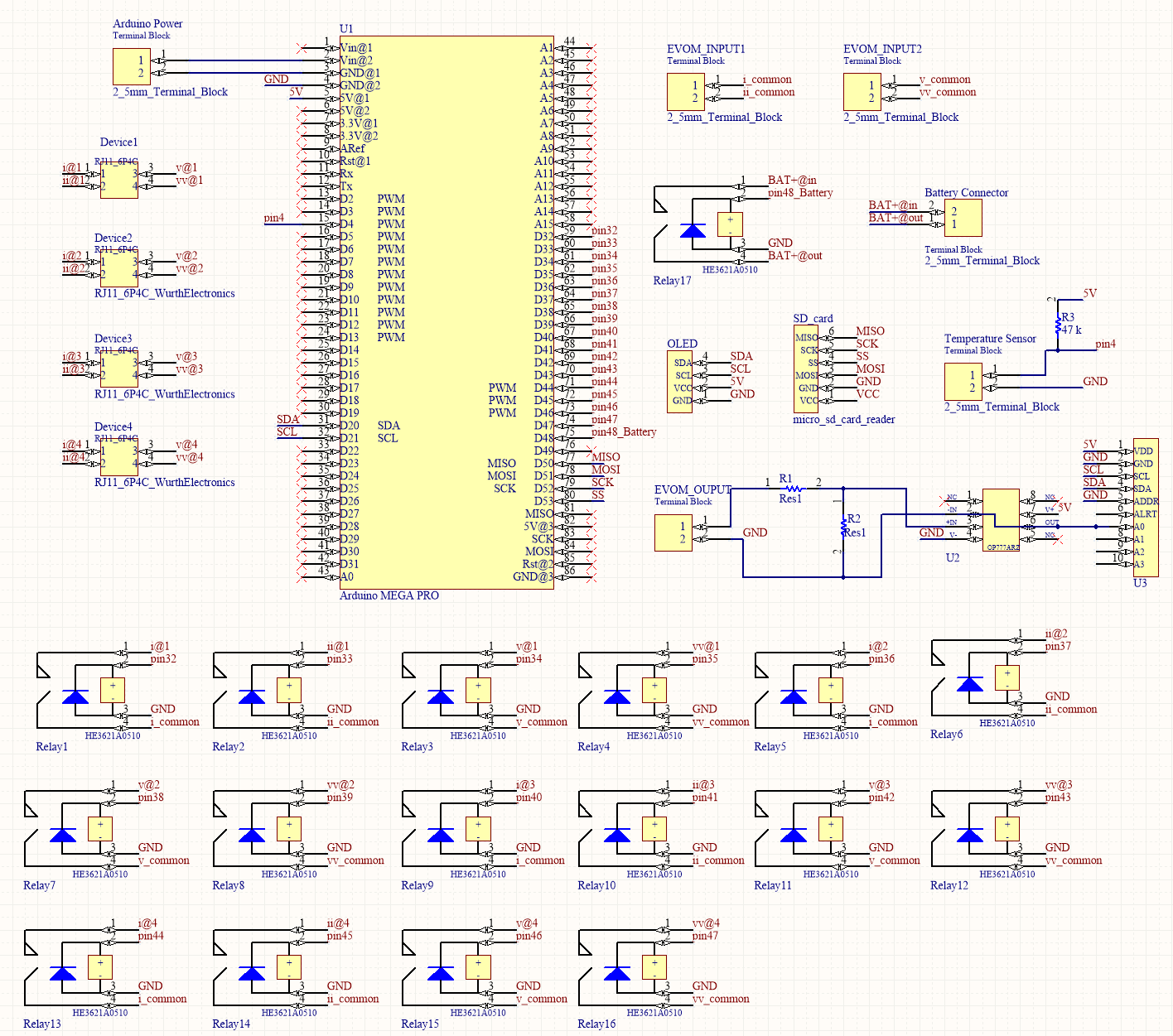


Figure S3. Schematic drawing of the electrical connections of the add-on-device. Please refer to Supplementary File 2 for the schematic drawing file.


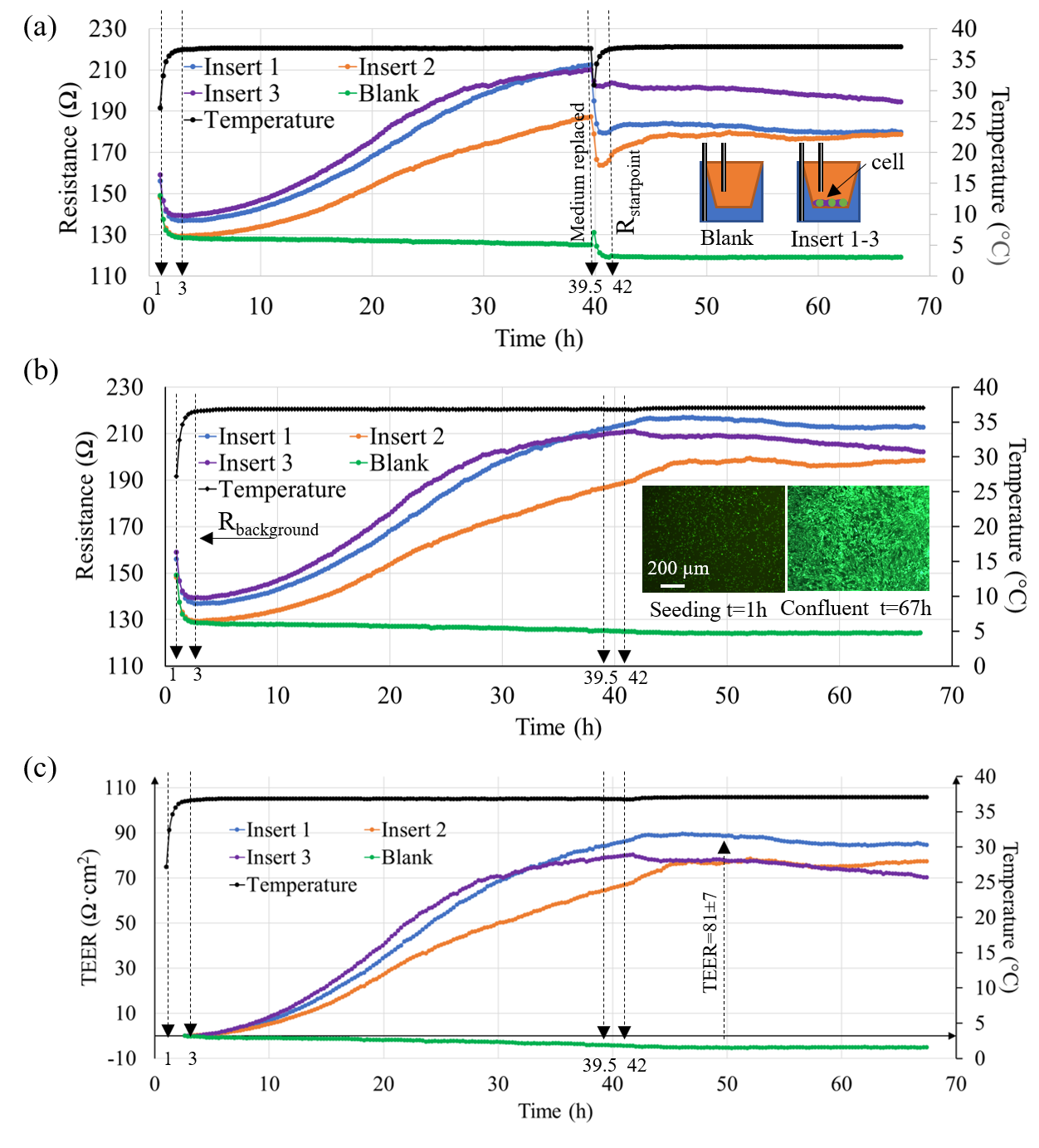


Figure S4. Real-time TEER measurement of ARPE-19 cells in transwell with in-situ temperature sensing: (a) Total resistance (raw data) is influenced by temperature change between room temperature and incubator temperature and electrode movement during medium change (b) the corrected data after medium change. Image of a sample shows cells 1h after seeding, and 67h when fully confluent. (c) Normalized TEER data after subtracting the background value at t=3h.


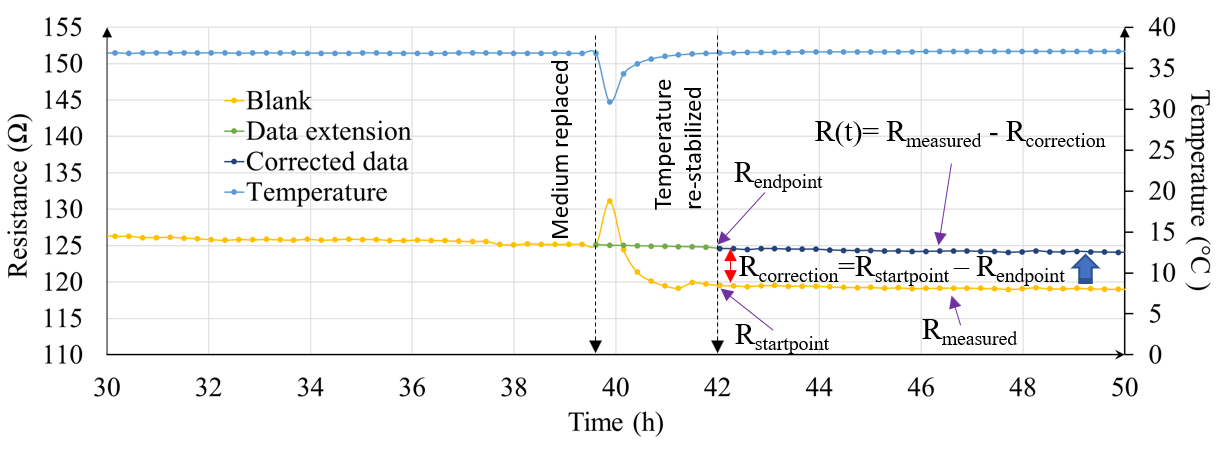


Figure S5. A copy of Figure 6 for further illustration of data correction after medium was replaced. Only “blank” data (t=30h to t=50 h) is plotted for clarity.

To compensate for disturbance in resistance value due to change in temperature, and electrode’s position, the data after medium change (until the temperature settled back to 37 ^o^C) were filled by extending the data trend (in excel highlight the data to be extended and drag them to empty cells to be filled automatically) before medium change. As shown in Figure. S5, the end point value obtained by the extension of the trend line, Rendpoint, was subtracted from the actual value recorded at that time, Rstartpoint, to calculate the correction factor, Rcorrection. The resistance values obtained after that, R(t), calculated by subtracting the measured value at that time, Rmeasured, to Rcorrection, was used for plotting the second half, and was calculated as:

R(t)= Rmeasured - Rcorrection

where Rcorrection=Rstartpoint – Rendpoint

Next, in order to obtain TEER value, subsequent values of each sample were subtracted from the background value. Here, the background was defined as the resistance value when temperature stabilized to 37 ^o^C at the beginning of the experiment.
